# Investigation of the Strain–Stress Field in Nanoscale Multilayer Systems by the Phase Plane Method

**DOI:** 10.3390/ma17102466

**Published:** 2024-05-20

**Authors:** Dmitrii Belous, Anna Badalyan, Alexei Khomenko, Alexander Goncharov

**Affiliations:** 1 Department of Applied Mathematics and Complex Systems Modeling, Sumy State University, 40007 Sumy, Ukraine; o.khomenko@mss.sumdu.edu.ua (A.K.); o.goncharov@mss.sumdu.edu.ua (A.G.); 2 Institute of Materials Science, Slovak University of Technology in Bratislava, Jána Bottu 25, 917 24 Trnava, Slovakia

**Keywords:** multilayer surface coating, nanoscale structure, strain–stress field, phase plane method

## Abstract

This paper presents the results of the study of stress relaxation fields, deformation, and temperature of the system of nanostructured multilayer coatings. In the work, a nonlinear relationship between strain and stress was used to take into account nonlinear effects in the mechanism of nanostructure formation. The paper assumes that a friction surface is provided by the self-organization of shear components: both stress and strain on the one hand, and temperature on the other. The studied objects are described in the adiabatic approximation, taking into account the fact of the evolution of stresses and strains. With the help of phase portraits of the system, the dependence of the deformation processes on the stresses arising in the system without coating and with coating is shown. It is shown that the rate of change of deformation depends on the characteristics of the mechanical impact on the coating and on the amount of stress and deformation. A conclusion is drawn regarding the transition process in the presence of two regions (Hooke and plastic deformation) in the corresponding phase portrait of the strain–stress field of the system. The results of the work can be used to determine the effective parameters of a coating in the analysis of experimental time dependences of stresses.

## 1. Introduction

Researching the properties of modern nanoscale multilayer systems, which include nanostructured coatings applied to the contact surfaces of technological equipment elements, is an urgent problem in modern surface engineering [1,2]. Operational characteristics of coated objects are determined by physical and technical characteristics, as well as the temperature distribution in the coating area and its effect on the internal stress–strain state of the structure of the near-surface volume of the material. In [3], an attempt was made to improve the performance and service life of a tungsten carbide (WC)-coated aluminum chromium nitride (AlCrN) tool by changing the properties of the applied coating. A specific effect of the surface layers of the applied coating on the physical and mechanical properties of the solid body was observed by modifying the surface layers, which made it possible to purposefully influence the processes of macroscopic deformation of materials. One study [4] presents an analysis of the synthesis process of thick PVD (Cr,Al)ON coatings and offers conclusions that allow us to understand how various process parameters affect the physical and mechanical characteristics of such objects. Another paper [5] presents a study of the evolution of temperature generation during hard machining of AISI 52100 steel with cutting tools made of Al_2_O_3_/TiCN mixed ceramics without coating and PVD with AlTiN coating. A significant reduction in cutting zone temperature was shown when machining with PVD-AlTiN-coated cutting tools compared to uncoated cutting tools in each experiment. The main reason for the lower temperature for AlTiN-coated tools is the lower coefficient of friction provided by the coating material compared to uncoated cutting tools, the authors believe. In addition, the excellent wear behavior of the AlTiN coating resulted in lower cutting temperatures.

It was established [6,7] that the effect of thermal load on the thermal barrier coating leads to the occurrence of compressive stresses and deformation transformations. This is explained by the fact that, with short-term thermal exposure, the multilayer structure of the coating manages to warm up and tends to expand, while the unheated substrate does not experience significant thermal impact in such a short time. Under the action of the resulting excessive compressive stresses and under the influence of deformation processes occurring in the coating, it changes, or may completely lose its physical and technical characteristics. The deformation processes of the multilayer coating, in turn, lead to a change in the strain–stress fields of the substrate.

Modification of the properties of metal surfaces that are under the influence of high-energy flows is effectively solved by applying multilayer nanostructured coatings [8,9,10]. Understanding the special role of the surface layer in the friction process provokes the interest of scientists in its research. In recent years, an approach to the study of deformation processes in surface layers has been actively developed based on the concept of structural levels of deformation and destruction of solid bodies, as well as on the understanding within this concept of the special role of the surface and surface layers of coatings in the initiation and development of deformation processes [11,12,13]. With the advent of new materials and strengthening technologies, the specific load at the points of contact of materials is constantly increasing. Accordingly, the temperatures reached on the friction surface also increase. According to many modern authors [14,15,16], the most important elements of the friction process are temperature effects and, as a result, deformation processes caused by temperature dynamics are observed in the near-surface layers. 

All of these described physical phenomena require effective research methods that would provide the possibility of theoretical analysis and prediction of strain–stress processes in a multilayer system under the influence of thermal loads. Modern numerical methods of studying deformation processes in multilayer systems provide for the construction of adequate mathematical models for the study of processes occurring in a wide range of temperature changes and deformations, as well as an adequate description of the behavior of the numerical solution in the solution domains. For the mathematical description of the analyzed processes, a complete dynamic system of partial differential equations of the hyperbolic type of the mechanics of deformed medium is often used. For its numerical solution, the mesh-characteristic method, adapted to the calculation of processes with a pronounced wave character, its hybrid variations and the smooth particle method, is used. These methods, along with their advantages, have significant disadvantages, which are contained in the fact that they do not form an idea of the behavior of the entire dynamic system with several components at the same time. At the same time, the phase space method forms a set of trajectories that define a set of groups of initial conditions and presents a graphical solution for the system’s differential equations, describing the state of the system. Phase trajectories give a complete picture of the nature of the processes in the system. However, the method of phase trajectories was not used until recently to study the dynamics of deformation effects in systems with a multilayer coating. The purpose of this work is to study the dynamics of deformation effects in multilayer structures using phase portraits and to analyze and compare theoretical conclusions for different surface structures.

Therefore, in Section 3, we define the dominant Lorentz-type evolution equations for the relaxation of strain and stress fields, as well as temperature. This model allows us to describe the material-dependent nanostructuring process of the surface. In contrast to previous approaches, the nonlinear relationship between strain and stress is used here to account for nonlinear effects in the nanostructure formation mechanism. The main assumption of our approach is that the surface in friction is provided by the self-organization of shear components of both shear stress σ and strain ε on the one hand and temperature *T* on the other. The relationship between the components σ and ε is well known, and the simplest case is described by the Kelvin–Voigt model. The temperature effect is caused by a critical increase in the shear modulus *G*(*T*) with decreasing temperature. The governing equations are obtained in Section 3.1, taking the above situation into account. In Section 3.2, we present a steady-state analysis of the principal ratios, which allows us to depict the formation of steady-state nanostructures. The description of the obtained nanostructures is done in the adiabatic approximation, taking into account the fact of stress and strain evolution. We obtain an expression for the Lyapunov exponent that determines the steady-state stability. In Section 3.3, two-dimensional phase portraits are used to illustrate the formation mechanisms of various nanostructures. In Section 4, the kinetics of the system are investigated, following the evolution of the strain and its rate of change. In Section 5, a brief conclusion is given. 

The relevance of the work lies in the fact that the most destructive process of thermomechanical impact on the coating material was investigated, i.e., the physical process of transition from elastic to plastic deformation in the structure of the coating. Previous studies conducted by scientists did not simultaneously cover several parameters in dynamics, such as strain, temperature, and stress, which lead to changes in the “strain–stress” fields of the substrate, and there were no attempts to observe the corresponding evolutionary processes and analytically explain the situation.

## 2. Temperature Influence on the Processes of Formation of Stress–Strain Fields

Solving the problem of changing the physical and technical characteristics of the surface layer of the material and establishing the features of the formation of stress–strain fields under the influence of external factors should be based on understanding the leading role of plastic deformation during friction, and the factors affecting it. Among these, first of all, one should include thermal action, which is caused by the transformation of mechanical energy. Thus, under the influence of heat flow in such structures, phenomena occur that cause phase and structural transformations, sometimes the destruction of the system. In [17], the effect of multilayer coatings on the thermal conductivity of tool samples with different coatings (without coating, TiN, TiN/TiCN, and TiN/Al_2_O_3_/TiCN) showed variations in temperature distribution due to the effect of coatings. TiN/Al_2_O_3_/TiCN was found to be the optimal tool coating (lowest thermal conductivity) of the four types. 

The inhomogeneity of the distribution of the thermal field in a solid body with multilayer structures applied to its surface was investigated in our works [18,19]. It was established that the multi-layer coating provides functions related to shielding or blocking of heat flows from frictional heat sources of the tool, affecting the characteristics of friction between surfaces, changing the parameters of plastic deformation processes, etc. It was established that the barrier coating reduces the coefficient of friction, and then the intensity of the heat flow decreases. The decrease in temperature in the study area occurs due to the reduction of the heat flow from friction in the cutting area, which is caused by the lower coefficient of friction of the workpiece–cutter pair in the case of a coated cutting plate. Such conclusions are in good agreement with the results of the research in works [20,21].

In [22], AISI4140 steel with different multilayer coatings (thin layers of Cr/CrN/CrAlN, CrN/CrAlN, and Cr/CrN that were applied by the PVD method) of different thicknesses were studied to determine thermal properties. The authors developed an empirical equation for the change in thermal properties with temperature. The thermal conductivity of the single-layer CrAlN system was lower than that of the multilayer structures. It is concluded that CrAlN and Cr/CrN/CrAlN coatings, due to their low thermal conductivity, can be used as a thermal barrier on the tool surface and thus can minimize the alternating internal stresses that cause substrate cracks.

## 3. The Phase Space Method in the Study of Strain–Stress Fields

One effective method of theoretical and experimental research into deformation processes is the analysis of phase portraits of physical phenomena [23,24]. The method of phase trajectories (phase space or plane) is a graph analytical method for the approximate study of nonlinear systems. The essence of the method is to evaluate the behavior of the system using visual geometric representations (phase parameters). The study of deformation effects using the method of phase portraits gives the most complete information and allows us to see the entire physical process at a glance. 

The study of the dynamics of deformation effects in strain-sensitive semiconductor films using the phase plane method is presented in [25]. The corresponding phase portraits showed the structural changes in the coating in the form of a break in the phase trajectory in the “resistance–deformation” plane. With periodic repetition of mechanical impact, the phase trajectory will turn into an open helical line. Using the phase plane method, assuming that one of the degrees of freedom has the highest relaxation rate, phase portraits of the boron-containing Al-C-B coating system were simulated in [26] and the diffusion process was studied. It was shown that near the stationary points in the phase portraits, either a slowing down of the evolution or a spiral twisting of part of the diffusion process is observed.

### 3.1. Basic Equations

The configuration for which we present a solution is a nano-sized multilayer system containing nanostructured coatings. It is widely accepted that the relaxation of the shear component of the strain tensor *ε* in nano-sized multilayer systems is determined by the Kelvin–Voigt equation [27,28]:

(1) $$\dot{\varepsilon }=-\varepsilon /\tau_{\varepsilon }+\sigma /\eta_{\varepsilon };$$

where *τ_ε_* is the Debye relaxation time and *η_ε_* is the effective shear coefficient. The second term on the right-hand side represents the deformation due to the action of the shear component of the stress σ. In stationary state, $\dot{\varepsilon }$ = 0 and Equation (1) is reduced to the Hooke-type relationship *σ* = *G_ε_ε*, where *G_ε_* ≡ *η_ε_*/*τ_ε_* ≡ *G*(*ω*)|*_ω_*_→0_ is the relaxed value of shear modulus (*ω* is circular frequency of a periodic external effect). The visco-elastic Kelvin–Voigt model (1) is used for metallic materials such as silicon iron Fe + 3% Si, mild steel, aluminum, and amorphous alloys Fe_40_Ni_40_B_20_ [29].

In the framework of phenomenological Landau theory, the phase transition is governed by the effective free energy *F*, which expands to a power series on *σ* [28,29,30,31,32,33,34,35,36,37]:

(2) $$F=F_{0}-\sigma \varepsilon =\frac{1}{2G\left(T\right)}\sigma^{2}+\frac{A}{4}\sigma^{4}-\sigma \varepsilon ,$$

where *G*(*T*) ≡ *G*(*ω*)|*ω*→∞ is the temperature-dependent unrelaxed shear modulus, *σε* means the external field *ε* effect, and *A* is a positive anharmonic constant. The equilibrium value of *σ* is determined by the following equation:

(3) $$\partial F_{0}/\partial \sigma =\varepsilon ,$$

where *F*_0_ is free energy at *ε* = 0. The relaxation transition to equilibrium state is described by the Landau–Khalatnikov-type equation [30,31,32,33,34,35,36]:

(4) $$\dot{\sigma }=-\frac{G{\left(T\right)}^{2}}{\eta }\left(\frac{\partial F_{0}}{\partial \sigma }-\varepsilon \right).$$

Here, *η* is a kinetic coefficient, which has the meaning of the shear viscosity. If σ is close to its equilibrium value *σ*_0_ = 0, the linear approximation *∂F*_0_*/∂σ* ≈ *σ*/*G*(*T*) can be used, where *G*(*T*) ≡ *∂σ/∂ε* = (*∂*^2^*F*_0_/*∂σ*^2^)^−1^. Hence, the relaxation Equation (4) presumes the linear form of the following:

(5) $$\tau_{\sigma }\dot{\sigma }=-\sigma +G\left(T\right)\varepsilon .$$

Here, the first term on the right-hand side describes the relaxation during time *τ_σ_* ≡ *η*/*G*(*T*). In a steady state $\dot{\sigma }$ = 0, the kinetic Equation (5) has the form of the Hooke’s law:

(6) $$\sigma =G\left(T\right)\varepsilon .$$

Substituting *ε*/*τ_σ_* for *∂ε/∂t* in Equation (5) reduces it to a Maxwell-type equation for a coating [28].

Note that the effective viscosity *η_ε_* ≡ *τ_ε_G_ε_* and a relaxed modulus *G_ε_* ≡ *η_ε_*/*τ_ε_* do not coincide with the real viscosity *η* and non-relaxed modulus *G*(*T*), respectively. This is caused by a different physical meaning of the Landau–Khalatnikov-type (5) and the Kelvin–Voigt (1) equations [27,28]. The values *G_ε_*, *η*, and *η_ε_* very weakly depend on the coating surface layer temperature *T*, while the shear modulus *G*(*T*) vanishes when the temperature decreases to *T_c_* [34]. Further, the temperature dependencies are used for the approximation: *G_ε_*(*T*), *η*(*T*), *η_ε_*(*T*) = const,

(7) $$G\left(T\right)=G_{0}\left(T/T_{c}-1\right),$$

where *G*_0_ ≡ *G*(*T* = 2*T_c_*) is a typical value of modulus. In order to present the self-organization process [31], the kinetic equation for temperature *T* is needed for completing the equations system (1) and (5), which contain the order parameter *ε*, conjugate field *σ*, and control parameter *T*. Employing the approach in [34], based on the elasticity theory relationships in [28], the following equation can be derived:

(8) $$c_{p}\dot{T}=\kappa \nabla^{2}T-\sigma \varepsilon /\tau_{\varepsilon }+\sigma^{2}/\eta_{\varepsilon },$$

where *c_p_* is the heat capacity and κ is the heat conductivity. The last term on the right-hand side stands for dissipative heating of a coating, flowing under the effect of the stress *σ*, that can be neglected in the case under consideration. 

On the other hand, the one-mode approximation (κ/*l*^2^)(*τ_T_Q*−*T*) ≈ κ∇^2^*T* can be used with acceptable accuracy in Equation (8) [28,31,34]. Thus, we consider the thermal effect of friction surfaces whose value is not reduced to the Onsager component and are fixed by external conditions (*l* is the scale of heat conductivity, i.e., the distance into which heat penetrates the coating, *τ_T_* ≡ *l*^2^*c_p_*/κ is the time of heat conductivity):

(9) $$\tau_{T}\dot{T}=\left(\tau_{T}Q-T\right)-\frac{l^{2}\sigma \varepsilon }{\kappa \tau_{\varepsilon }},\cdots Q=Q_{0}+\sigma^{2}/c_{p}\eta_{\varepsilon }.$$

Here, *Q_0_* is a heat flow from the surrounding solids to the surface layer. The square contribution of the stress is implied to be included in the rubbing surfaces temperature *T_e_* = *τ_T_Q*. The obvious account of this term leads to a significant complication of the subsequent analysis, though it results only in renormalization of the quantities. Therefore, for our further consideration, the component *T_e_* = *τ_T_Q* in Equation (9) is presumed to be constant. It is noteworthy that during derivation of Equation (9) we accepted the equilibrium value of the temperature of surface layer *T*_00_ to be equal to zero. Evidently, contrary to the surface being heated initially to the temperature *T*_00_ ≠ 0, the term *T*_00_/*τ_T_* should enter Equation (9). This term describes the relaxation of the current temperature of the surface layer to its equilibrium value *T*_00_ in the absence of the heat flow *Q* from the background solids. It is convenient to introduce the following measure units:

(10) $$\sigma_{s}={\left(c_{p}\eta_{\varepsilon }T_{c}/\tau_{T}\right)}^{1/2},\;\;\;\;\;\;\;\varepsilon_{s}=\sigma_{s}/G_{\varepsilon },\;\;\;\;\;\;\;\;\;\;\;T_{c}$$

for the variables *σ*, *ε*, and *T*, respectively. Then, the basic Equations (1), (5) and (9) are reduced to a form applicable to any viscoelastic medium [31,34].

### 3.2. Determination of Coordinates of Special Points of the System

According to the synergetic concept of consideration of plastic deformation processes’ changes of strains and stresses, density of defects do not behave autonomously, but in a self-coordinated manner. At the phenomenological level, such behavior is described by a system of differential equations that contain nonlinear terms. The solution of such systems is effectively represented graphically in the form of phase portraits [35,36,37,38,39]. 

The model we take as a basis is a surface film structure to strengthen and increase the thermal stability of a cutting tool. Let us write a system of dimensionless equations for the analysis of this model [39]:

(11) $$\tau_{\varepsilon }\frac{d\varepsilon }{dt}=-\varepsilon +\sigma ,$$

(12) $$\tau_{\sigma }\frac{d\sigma }{dt}=-\sigma +g(T-1)\varepsilon ;$$

(13) $$\tau_{T}\frac{dT}{dt}=(T_{e}-T)-\sigma \varepsilon .$$

Here the constant *g* = *G*_0_/*G_e_*, relaxation times of stress *τ_σ_*, temperature *τ_T_*, and strain *τ_ε_* are introduced, and temperature *Te* is the temperature away from the cutting surface, i.e., the thermostat, the constant *g* < 1. These equations formally derive from the synergetic Lorentz system, in which the role of the order parameter is played by the strain, the conjugate field reduces to stress, and temperature is the control parameter. 

We will use the phase plane method, which allows us to determine the phase portraits of the system. Their exact form is found by numerical integration of the equations using the Runge–Kutta method of the 4th order of accuracy. In fact, the system of Equations (11)–(13) is dimensionless except for measurable time. Since *τ_T_* << *τ_ε_*, *τ_σ_*, then in (13) we can put *dT/dt* = 0, which gives the relation as follows:

(14) $$T=T_{e}-\varepsilon \sigma$$

Substitution of (14) in (11), (12) leads to the system as follows:

(15) $$\tau \frac{d\varepsilon }{dt}=-\varepsilon +\sigma ,$$

(16) $$\frac{d\sigma }{dt}=-\sigma +(T_{e}-\varepsilon \sigma -1)g\varepsilon ;$$

where $\tau_{\sigma }$ is a scale to measure time and parameter $\tau \equiv \tau_{\varepsilon }/\tau_{\sigma }$. 

To determine the stable states of the system from the point of view of the phase plane method, it is necessary to find the coordinates of special points. Dividing (15) by (16), we find the singular points of the phase plane, that is, the points in which the direction of the tangent to the phase trajectory is not defined. To do this, we will write a system of equations:

(17) −ε+σ=0,

(18) $$\tau^{-1}\left[-\sigma +(T_{e}-\varepsilon \sigma -1)g\varepsilon \right]=0.$$

**Figure 1:**
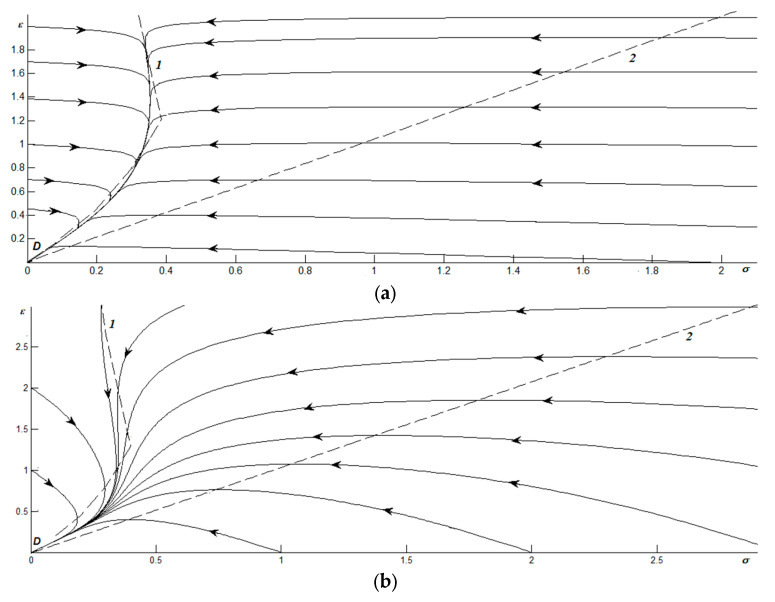

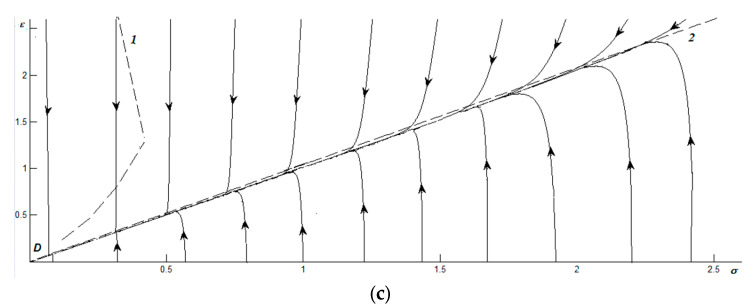
Phase portraits for *g* = 0.5, thermostat temperature *T_e_* = 2, and relationships of relaxation times as follows: (**a**) $\tau_{T}<<\tau_{\varepsilon }=0.01\tau_{\sigma }$; (**b**) $\tau_{T}<<\tau_{\sigma }=\tau_{\varepsilon }$; (**c**) $\tau_{T}<<\tau_{\varepsilon }=100\tau_{\sigma }$.

**Figure 2:**
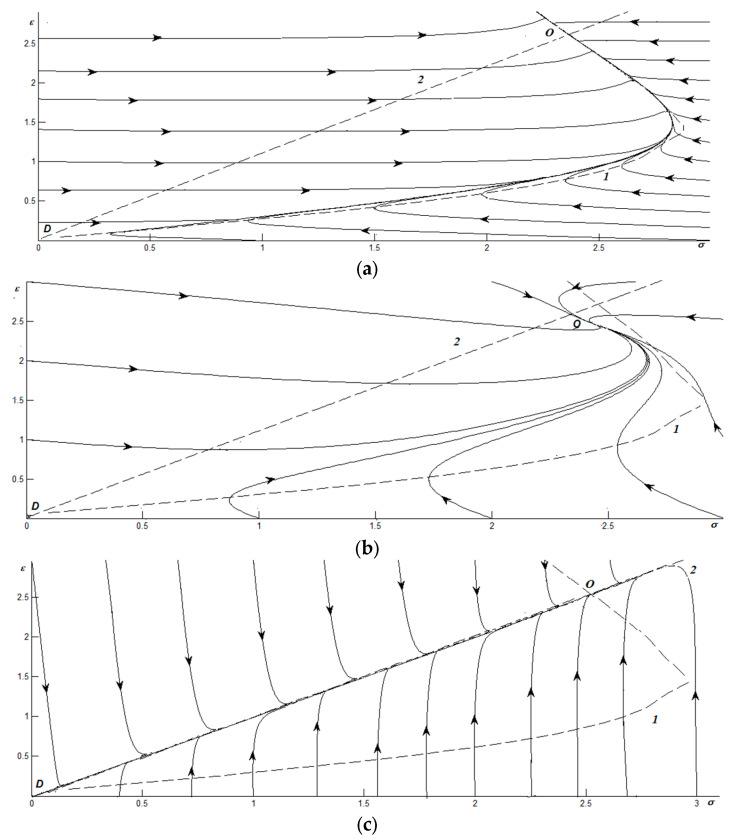
Phase portraits for *g* = 0.5, thermostat temperature *T_e_* = 9, and relationships of relaxation times as follows: (**a**) $\tau_{T}<<\tau_{\varepsilon }=0.01\tau_{\sigma }$; (**b**) $\tau_{T}<<\tau_{\varepsilon }=\tau_{\sigma }$; (**c**) $\tau_{T}<<\tau_{\varepsilon }=100\tau_{\sigma }$.

The phase portrait has special points D(0,0), $O\left(\sqrt{T_{e}-\left(g^{-1}+1\right)},\sqrt{T_{e}-\left(g^{-1}+1\right)}\right)$ (see Figure 1 and Figure 2), the second of which is realized only in the area of fulfillment of the condition $T_{e}>T_{c0}=1+g^{-1}$. The corresponding Lyapunov exponents have the form as follows.

(1) For point *D* (0,0), the Lyapunov exponent has the form as follows:
  (19) $$\lambda_{D}=-\frac{1}{2}\left[1+\tau^{-1}\right]\left\{1\pm \sqrt{1-4\tau^{-1}\left(1+g(T_{e}-1)\right)}\right\}.$$
(2) For the point $O\left(\sqrt{T_{e}-\left(g^{-1}+1\right)},\sqrt{T_{e}-\left(g^{-1}+1\right)}\right)$, the form is as follows:
  (20) $$\lambda_{o}=-0.5\left(1+\tau^{-1}\right)\left[1\pm \sqrt{1-8\tau \left(g^{-1}-1\right)\left[T_{e}-g^{-1}-1\right]}\right].$$

From this we can see that for values of the parameter τ bounded from above by the value are as follows:

$$\tau_{c}=\frac{\left(g^{-1}-1\right)\left[T_{e}-g^{-1}-1\right]}{8}$$

point *O* gives a stable node, and with its growth to values $\tau >\tau_{c}$ represents a focus.

### 3.3. Phase Portraits of the System

According to experimental data for cutting materials, the stress relaxation time is $\tau_{\sigma }$~10^−10^ s. The temperature relaxation time to the value *T_e_* satisfies the condition as follows:

(21) $$\tau_{T}<<\tau_{\sigma },\tau_{\varepsilon }.$$

As a result, for the obtained two-parameter system (15), (16), the obtained phase portraits are shown in Figure 1 and Figure 2.

Figure 1 shows the phase portraits describing the behavior of the system in the dry friction mode (for the temperature of the friction surfaces lower than *T_c_*_0_), for different ratios of the relaxation times *τ*. In particular, Figure 1a corresponds to the example of τ = 0.01. Dashed lines 1 and 2 show the isoclines obtained when equating the derivatives in Equations (15) and (16) to zero, respectively. Therefore, curve 1 corresponds to system parameters under which the stress does not change, and line 2 corresponds to an example of preservation of strain. These lines intersect at the origin, forming a single stationary point *D*, which is a node. It can be seen that the phase trajectories converge to node *D*, i.e., over time the stress relaxes to a zero value. Moreover, during movement along the phase plane under arbitrary initial conditions, two stages are observed: in the first, there is an instantaneous relaxation of the system to a line close to isocline 1; in the second, a slow movement along the specified curve occurs. At the first stage, the strains are preserved, which resembles the previously described transition between friction modes. Note that the line along which the system moves in the second stage corresponds to the Hooke’s section of dependence σ(ε). Thus, when *ε* = 0, sticking occurs, and when *ε* ≠ 0, sliding occurs. 

The phase portrait shown in Figure 1b is constructed for an example when the relaxation times of stress and strain coincide (*τ* = 1). It is also characterized by a special point *D*, which is a node. Here, situations are possible when the strain ε first increases and then decreases, and vice versa. This means that by the time the system reaches equilibrium (origin of coordinates), discontinuous motion is possible. For example, according to the phase trajectories starting at *ε* = 0, first the dry mode of friction is realized (stress is 0), then sliding begins (stresses increase), and then dry friction takes place again. The most complex type of discontinuous motion is described by phase trajectories that are tangent to the isocline 1. Here the system behaves as follows: the stress first increases, then decreases (after the first crossing of isocline 1), then increases again (after the second crossing), and finally relaxes to zero (after the third and last crossing). 

Figure 1c corresponds to the example of *τ* = 100. Here, as in Figure 1a, two stages are distinguished: a fast relaxation to a line close to isocline 2, and then a slow movement along it. At the first stage, the stress changes little, and the strain decreases very quickly if their initial values of ε are above isocline 2, or it increases if their initial values are below isocline 2. At the second stage, in the upper part of the phase portrait, the configuration point moves along the plastic section and then moves along the elastic section. In the last section, the system stays for a longer time, since it is closer to the isocline than the first.

Figure 2 shows phase portraits for the same parameters and relaxation time ratios as in Figure 1, but at a temperature corresponding to the plastic deformation region. Here, sliding friction is realized during mechanical impact in the solution zone and, over time, a non-zero stationary value of the shear strain *ε*_0_ ≠ 0, which corresponds to the minimum of the potential, is established. As before, lines 1 and 2 are isoclines of phase trajectories. Phase portraits are characterized by two special points at saddle *D* at the origin of coordinates, and a node *O* at the site of non-zero values of stress and strain, which are given by the intersection of isoclines. 

At *τ* = 0.01, the picture shown in Figure 2a is observed. Here, as in Figure 1a, the phase trajectories quickly converge to a line close to isocline 1 from any point of the phase plane under the condition that strain is preserved. Next, the system relaxes to a non-zero value *ε_0_* ≠ 0, and stationary sliding friction is established.

However, the straight line along which the movement is carried out in the second stage corresponds to the plastic section of the dependence σ(ε), that is, the system always slides, except for those situations when the initial stress values are close to zero. Note that, over time, sliding becomes more viscous if *ε_i_* < *ε*_0_, and conversely drier when *ε_i_* > *ε*_0_, where *ε_i_* and *ε*_0_ are the initial and stationary values of strains.

As can be seen from Figure 2b, for *τ* = 1, in the case of establishing a stationary value of stress and strain, the following situations are possible during which the plastic characteristics of the coating change over time and various intermittent modes of friction are realized. Figure 2c shows the phase portrait for *τ* = 100, where, as in Figure 1c, there are two stages. The main difference is that in the latter, the Hooke’s section is closer to the isocline, and in the case of evolution, the system is on it for more time than it is on the plastic one. Stationary point *O* is located in the plastic region.

## 4. Kinetics of the System Coordinates $\dot{\varepsilon }-\varepsilon$

Let us investigate the kinetics of the system, following the evolution of strains and the rate of their change. To do this, from two first-order differential Equations (15) and (16), depending on the stresses σ and strains ε, we obtain the second-order equation for ε. For this, it is necessary to express σ in terms of ε from (15) and write down the time derivative of this expression. Next, substituting the obtained dependencies $\sigma \left(\varepsilon ,\dot{\varepsilon }\right)$, $\dot{\sigma }\left(\varepsilon ,\dot{\varepsilon }\right)$ into (16), we find the desired equation:

(22) $$A\ddot{\varepsilon }+B\dot{\varepsilon }+C=0,$$

*A* = *τ, B* = *τ *(*gε*^2^ + 1) +1, *C* = *ε*[1 − *g*(*T_e_* − *ε*^2^ − 1)].

**Figure 3:**
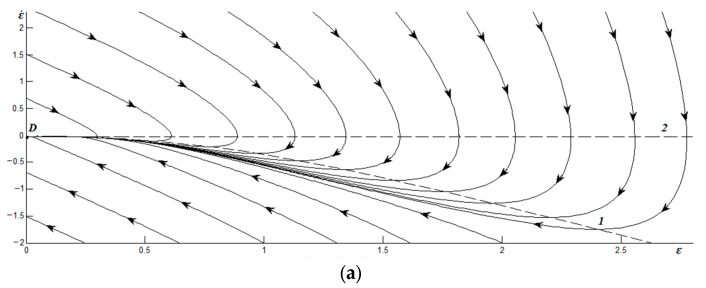

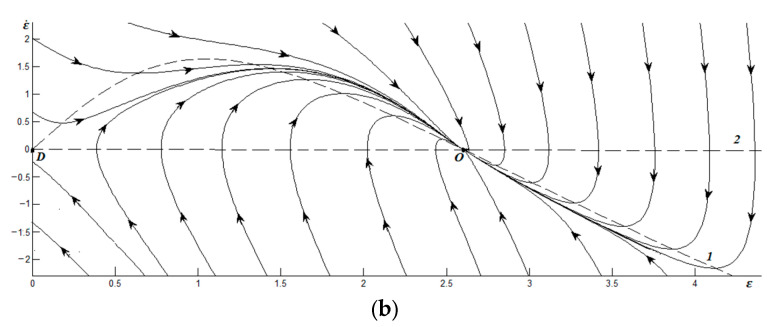
Phase portraits for relaxation times *τ_ε_* = *τ_σ_*, *g* = 0.5, and thermostat temperatures as follows: (**a**) *T_e_* = 2; (**b**) *T_e_* = 9.

This describes the reactive-dissipative mode, as it has the second and first order time derivatives. The corresponding phase portraits are presented in Figure 3. 

Here, the dashed curve 1 is an isocline on which the rate of strain change remains constant $\left(\ddot{\varepsilon }=0\right)$. Dashed curve 2 in Figure 3 corresponds to the isocline where the strains do not change $\left(\dot{\varepsilon }=0\right)$. Since there is no stress in Equation (22), their initial values are given by $\varepsilon ,\dot{\varepsilon }$.

Figure 3a shows the phase portrait corresponding to the temperature of the friction surfaces below the critical *T_c_*_0_. Here, the relaxation of *ε* to the node *D* at the origin is observed. According to the phase trajectories, intermittent sliding is also possible at the same time.

The phase portrait shown in Figure 3b corresponds to an example when *T_e_* is higher than *T_c_*_0_ and sliding occurs. It is characterized by two special points: a saddle *D* and a stable node *O*. Over time, under arbitrary initial conditions, the system reaches a steady state corresponding to point *O*, and the strain does not change thereafter $\left(\dot{\varepsilon }=0\right)$. It can be seen that strain relaxation can occur in the presence of intermittent friction.

Estimated values of possible strains are currently quite difficult to provide, because experimental work in this area is limited to expressions like “…further deformation occurs due to stress and temperature…”. Therefore, this work includes studies in the entire range of stress, strain, and temperature values. However, we can unequivocally state that the presence of deformation should lead to an intermittent mode of friction since it is observed experimentally. All of these parameters are effective and are very different from the coverage parameters. Currently, they are not all measured, and in experimental works they are not given in full. That is, we present work that can be used to determine the effective parameters of a coating in the analysis of experimental time dependencies of strains. After that, it will be possible to find the strain values that are implemented in the system. However, for this it is necessary to conduct specific experiments. Moreover, a qualitative picture of the obtained results is illustrated by figures, which show the time dependencies of strain. Such dependencies are measured experimentally. There are also a number of experimental works in which it is shown that dry friction exhibits self-similar behavior (see, for example, references [16,17,18,19]). That is, the connection with the experiment is given numerically by calculating the time dependencies of stresses and concluding that they have a fractal structure. Recently, the method of multifractal analysis has been precisely used for the analysis of experimentally obtained time dependencies that have a stochastic nature.

## 5. Conclusions

The study of the strain–stress field in multilayer systems was carried out by forming a mathematical model of deformation processes, which is represented by a system of differential equations based on the Lorentz synergetic system.

The analysis of the phase portraits of the system in the friction mode showed the dependence of the deformation processes on the stresses arising in the system without a coating (so-called dry friction) and with a coating (movement along phase trajectories from a stationary point in the direction of increasing the corresponding indicators) under the action of the corresponding load, which is regulated by temperature gradient. The two isoclines of the phase portraits form the state of the system in which the stress and strain acquire a constant value, and the corresponding phase trajectories of the system describe the change in the stress–strain state of the studied objects around a stationary point (a node).

Different forms of phase portraits characterize the influence of mechanical processing parameters on the coating material of the cutting tool. An increase in temperature to plastic deformation indicators in a multilayer coating system forms a phase portrait related to stationary sliding friction. When studying the stationary value of stress and strain, a change in the plastic characteristics of the coating over time during the implementation of various intermittent modes of friction was established. A stationary point in the system characterizes the behavior of the material without a coating.

The study of the kinetics of the system, the analysis of the evolution of strain, and the rate of its change showed that the rate of change of strain depends on the characteristics of the mechanical impact on the coating and the influence of the corresponding stress and strain characteristics. The presence of two areas (Hooke’s and plastic deformation) on the corresponding phase portrait of the strain–stress field of the system establishes the characteristics of the transition of the system from one state to another and allows for a prediction of the corresponding deformation processes in the coating.

## Data Availability

Data are contained within the article.
